# Analysis of the molecular mechanism underlying di(2-ethylhexyl) phthalate-induced bladder carcinogenesis via network toxicology and molecular docking approaches: An observational study

**DOI:** 10.1097/MD.0000000000047378

**Published:** 2026-02-06

**Authors:** Manfei Jiang, Chuanwei Sun, Baofeng Wang, Qingmai Huang, Qianghua Hu, Xianping Che

**Affiliations:** a Department of Urology, The Second Affiliated Hospital of Hainan Medical University, Haikou City, Hainan, China; b Department of Breast and Thyroid Surgery, The Second Affiliated Hospital of Hainan Medical University, Haikou City, Hainan, China.

**Keywords:** bladder cancer, data mining, di(2-ethylhexyl) phthalate, molecular docking, network toxicology

## Abstract

This study aims to investigate the toxicity of di(2-ethylhexyl) phthalate (DEHP) and the potential molecular mechanisms of DEHP-induced bladder cancer (BLCA) using network toxicology and molecular docking strategies. The toxicity of DEHP was assessed using Prox-II software, and potential targets for DEHP-induced BLCA were identified by integrating data from ChEMBL database, Search Tool for Interactions of Chemicals, SwissTargetPrediction, GeneCards, Therapeutic Target Database, Online Mendelian Inheritance in Man, and The Cancer Genome Atlas. STRING database and Cytoscape were employed to construct target networks and determine core targets. The expression levels of core targets were analyzed using R. Gene Ontology and Kyoto Encyclopedia of Genes and Genomes pathway enrichment analyses were performed on potential and core targets. Molecular docking was carried out using CB-Dock 2 to verify the interactions between DEHP and core targets. A total of 105 potential targets related to DEHP-induced BLCA were identified, from which 7 core targets were selected: cyclin-dependent kinase 1, interleukin 6, cyclin-dependent kinase 2, cyclin B1, Erb-B2 receptor tyrosine kinase 2, cyclin B2, and B-cell lymphoma 2. IL-6 and B-cell lymphoma 2 showed downregulated expression in tumor tissues, while cyclin-dependent kinase 1, cyclin-dependent kinase 2, cyclin B1, Erb-B2 receptor tyrosine kinase 2, and cyclin B2 were upregulated. Gene Ontology and Kyoto Encyclopedia of Genes and Genomes enrichment analyses indicated that these targets were enriched in cell signaling and cancer-related pathways. Molecular docking confirmed that DEHP interacts with these core targets. DEHP may promote the development of BLCA by interacting with key proteins and signaling pathways. This study provides a theoretical basis for understanding the molecular mechanisms of DEHP-induced BLCA and offers references for future prevention and treatment strategies.

## 1. Introduction

The extensive use of plastics in recent decades has led to environmental accumulation, posing a threat to both ecology and human health. Sharma et al^[1]^ pointed out that plastic production and waste disposal contribute to greenhouse gas emissions and climate change, while microplastics interfere with marine carbon sequestration and health. Microplastics, as contaminants associated with plastics, pose significant risks to public health through various pathways. Microplastics can adsorb heavy metals and persistent organic pollutants, entering the food chain via soil, plants, and animals, thereby threatening human health.^[2]^ Rist et al^[3]^ found that the inhalation and clearance of pulmonary microplastics are influenced by factors such as hydrophobicity, surface charge, functionalization, protein corona, and particle size, which may lead to lung damage. Environmental pollution is also a risk factor for cancer, such as the association between industrial air pollution and ovarian cancer,^[4]^ and the close relationship between the incidence of bladder cancer (BLCA) and exposure to certain chemicals.^[5]^

Di(2-ethylhexyl) phthalate (DEHP), a commonly used plasticizer in plastics, belongs to phthalates and has carcinogenic and endocrine-disrupting effects on the environment.^[6,7]^ DEHP is widely present in plastic toys, water equipment, food packaging, flooring, personal care products, and medical devices. Zhang et al^[8]^ found that DEHP can migrate from water supply materials, emphasizing the use of DEHP in pipeline materials and plastic components related to water. Klotz et al^[9]^ highlighted the need to remove DEHP during polyvinyl chloride flooring recycling to ensure safety. This suggests that DEHP is a common additive in polyvinyl chloride flooring products and is widely found in residential and commercial environments. DEHP can be detected in air, industrial wastewater, soil, and river water,^[10]^ entering the human body through inhalation, ingestion, and skin contact, where it is metabolized into MEHP to exert its toxicity.^[11]^ Studies have shown that DEHP exposure is associated with cancer,^[12]^ and cross-sectional studies have found that it is linked to an increased risk of malignant tumors in the reproductive system.^[13]^ In vitro experiments have shown that DEHP and its metabolites can promote the proliferation of prostate cancer cells.^[14,15]^ New toxicity testing strategies in the 21st century emphasize computer models based on toxicity pathways to support in vitro testing and predict the potential toxicity of chemicals.^[16]^ Therefore, it is necessary to integrate and analyze the molecular mechanisms from different studies to identify key toxicological pathways for substances. Network toxicology, combining network pharmacology and network biology, uses multi-omics and database analysis to examine the relationship between chemical toxicity and its targets.^[17]^ Limited research has been conducted on DEHP in the bladder, but studies have shown that exposure to DEHP may increase the risk of bladder overactivity.^[18]^ However, there is still little research on the relationship between DEHP exposure and BLCA. This study uses network toxicology to explore the key molecular targets and toxicological pathways of DEHP-induced BLCA, providing a new strategy for disease prevention and environmental toxicity assessment, and laying the foundation for the implementation of 21st century toxicity testing strategies.

## 2. Materials and methods

### 2.1. Toxicity analysis of DEHP and target pooling

We utilized the ProTox-II software (https://tox.charite.de/protox3/index.php?site=home) to gather information on the toxicity induced by DEHP. The chemical structure and SMILES representation of DEHP were obtained by searching for “di(2-ethylhexyl) phthalate” in the PubChem (RRID:SCR_004284) database (https://pubchem.ncbi.nlm.nih.gov).^[19]^ We selected the species *Homo sapiens* by querying the ChEMBL (RRID:SCR_014042) database (https://www.ebi.ac.uk/chembl) to identify potential targets of DEHP.^[20]^ The ChEMBL IDs retrieved were then uploaded to the UniProt database for gene name correction and conversion.^[21]^ Additionally, the SMILES notation was submitted to the Search Tool for Interactions of Chemicals (STITCH) database (https://stitch.embl.de/) and the SwissTargetPrediction database (http://swisstargetprediction.ch) to further identify potential DEHP targets in “*Homo sapiens*.”^[22]^ Finally, we employed the “ggvenn” package in R (version 4.3.2; Virginia) to remove duplicates and merge the targets obtained from the 3 databases, thereby constructing a comprehensive target library for DEHP.

### 2.2. Collection of BLCA targets

The GeneCards (RRID:SCR_002773) database (https://www.genecards.org/) was searched for ‘bladder cancer’ and filtered based on a relevance score >10 to identify BLCA-related targets. Subsequently, searches were conducted in the Online Mendelian Inheritance in Man (OMIM; https://omim.org/) and Therapeutic Target Database (TTD; https://db.idrblab.net/ttd/) databases for BLCA-related targets.^[23]^ The targets retrieved from these 3 databases were then de-duplicated and consolidated using the R package “ggvenn” to generate a comprehensive list of target genes. Additionally, transcriptome RNA-seq data for the BLCA cohort were downloaded from the The Cancer Genome Atlas (TCGA) database. The data were organized using Strawberry Perl software (version 5.30.01; Virginia), and differential gene expression was analyzed using the R package “limma.” Expression thresholds were set with a *P*-value ≤ .05 and a log fold change (log FC) of 1 (a 2-fold difference). The resulting differentially expressed genes were further filtered and compared with the previously identified target genes. The intersection of these 2 gene sets was determined using the Venn 2.1.0 online analysis tool, yielding the final list of intersecting target genes. These intersected target genes were used to establish the target gene library for BLCA.

### 2.3. Construction of the regulatory network of DEHP targets and BLCA targets

Using the “ggvenn” package in R, we read the DEHP and BLCA target list files to obtain the network relationship files and node attribute files for the intersecting targets. These files were then imported into Cytoscape (RRID:SCR_003032) software (version 3.10.0) to construct the regulatory network. The intersecting targets were subsequently identified as potential targets for DEHP-induced BLCA.^[24,25]^

### 2.4. Construction of the protein interaction network and selection of core targets

We used the STRING database (https://cn.string-db.org) to construct a network of interactions between BLCA and DEHP targets. First, the potential targets of DEHP-induced BLCA were imported into the STRING database, with the species “Homo sapiens” selected, and the protein–protein interaction (PPI) network set to “Highest Confidence > 0.7.” The results generated by STRING were then imported into Cytoscape software to calculate the median network node degree. Centrality metrics for each node, such as maximum neighborhood component, degree, edge percolated component, bottleneck, closeness, and betweenness, were calculated using Cytoscape software. Targets exceeding the top 10 centrality values were sorted according to their centrality scores, and the top 4 genes for each centrality metric were selected as the core BLCA-inducible targets of DEHP. The differential expression of these core targets in tumor and normal samples was analyzed using the “limma” and “ggplot2” packages in R.

### 2.5. Gene function and pathway enrichment analysis of target proteins

We used the “enrichplot,” “org.Hs.e.g..db,” “ggplot2 (RRID:SCR_014601),” and “clusterProfiler” packages, with a filter condition set to *P* ≤ .05. Gene Ontology (GO) analysis and Kyoto Encyclopedia of Genes and Genomes (KEGG) pathway enrichment analysis were conducted on the potential targets of DEHP-induced BLCA to elucidate the primary biological functions and toxicity pathways of these targets.^[26-28]^ Additionally, KEGG analysis was performed on the core targets using the same method to further investigate the pathways associated with BLCA in these core targets.

### 2.6. Molecular docking of DEHP and its core targets

We retrieved the structure of the core target from the RCSB PDB database (https://www.rcsb.org/) to obtain the relevant PDB files.^[29]^ The core target protein structure data and DEHP ligand structure files were then imported into the online CB-Dock 2 database (https://cadd.labshare.cn/cb-dock2/php/index.php) for molecular docking and visualization.

## 3. Results

### 3.1. Toxicity analysis of DEHP and identification of induced BLCA targets

**Figure 1.**
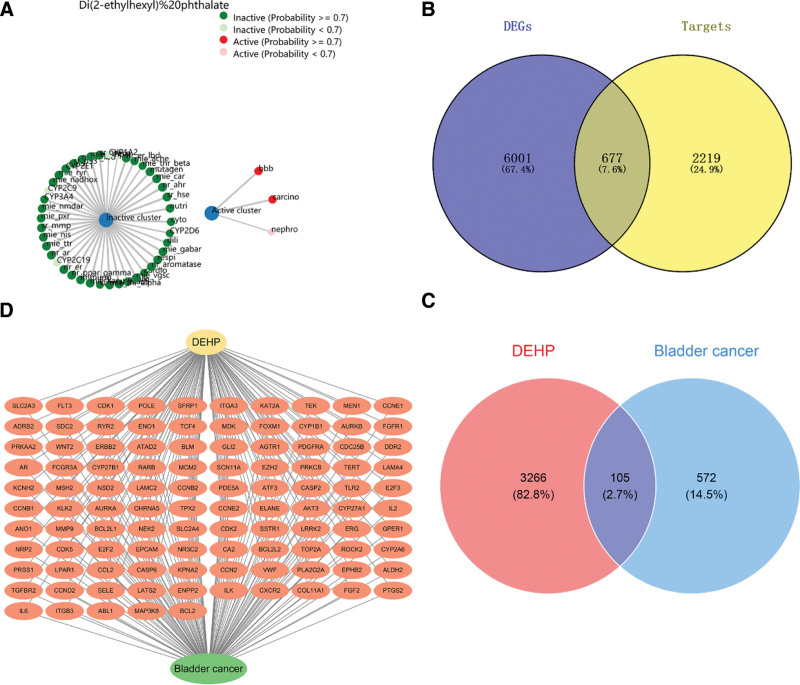
The toxicological profile of DEHP and its associated targets implicated in the induction of BLCA. (A) Toxicity analysis of DEHP, Toxicity targets: green, inactive; red, active. The redder the color is, the greater the likelihood of predicted toxicity. (B) The intersection of BLCA targets identified through the TCGA database in conjunction with GeneCards, OMIM, and TTD databases comprises 677 distinct entities. (C) The intersection of DEHP target points and BLCA target points includes 105 target points. (D) The image shows specific targets of the 105 potential target genes of DEHP-induced BLCA. BLCA = bladder cancer, DEG = differentially expressed gene, DEHP = di(2-ethylhexyl) phthalate, OMIM = Online Mendelian Inheritance in Man, TCGA = The Cancer Genome Atlas, TTD = Therapeutic Target Database.

We gathered information on the toxicity of DEHP through ProTox-II, which includes carcinogenicity, nephrotoxicity, and neurotoxicity (Fig. 1A). We identified 2896 targets strongly associated with BLCA using the GeneCards, OMIM, and TTD databases. Additionally, the TCGA database provided 6678 differential genes related to BLCA. By intersecting these 2 datasets, we identified 677 overlapping target genes, which were designated as the final target genes for BLCA (Fig. 1B). Subsequently, a total of 3371 DEHP-related targets were screened across the ChEMBL, SwissTargetPrediction, and STITCH databases. By intersecting the 3371 DEHP targets with the 677 BLCA-related targets, 105 common targets were identified (Fig. 1C). These 105 target genes represent potential DEHP-induced targets associated with BLCA (Fig. 1D).

### 3.2. Protein–protein interaction network and identification of core targets

**Figure 2.**
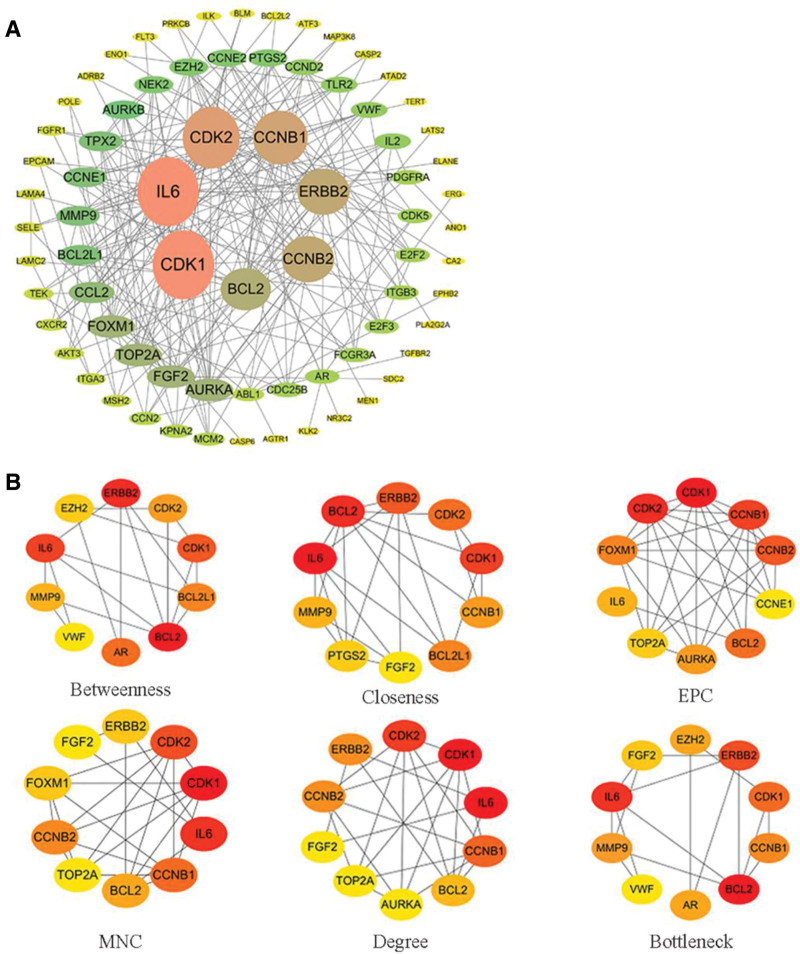
The core target induced by DEHP in BLCA. (A) Cytoscape software was used to construct a potential target PPI network; the larger the node is, the darker the color represents a higher degree value. (B) Evaluation of 6 topological parameters of the cytoHubba core target network. BLCA = bladder cancer, DEHP = di(2-ethylhexyl) phthalate, PPI = protein–protein interaction.

We constructed a PPI network using the STRING database, which comprises 105 nodes and 214 edges, resulting in an average node degree of 4.08 and a PPI enrichment *P*-value of <1.0e−16. Within this network, the nodes cyclin-dependent kinase 1 (CDK1), interleukin 6 (IL6), cyclin-dependent kinase 2 (CDK2), cyclin B1 (CCNB1), Erb-B2 receptor tyrosine kinase 2 (ERBB2), cyclin B2 (CCNB2), and B-cell lymphoma 2 exhibit degrees >10, with respective values of 18, 18, 16, 15, 14, 14, and 13 (Fig. 2A). Subsequently, we identified CDK1, IL6, CDK2, CCNB1, ERBB2, CCNB2, and BCL2 as core targets within the PPI network. This was achieved through the evaluation of topological parameters, including maximum neighborhood component, degree, edge percolated component, bottleneck, closeness, and betweenness, using the cytoHubba plugin in Cytoscape. The color intensity of nodes corresponds to the degree of connection. The redder the node is, the greater the degree of connection. The top 4 gene core targets with the reddest color for each topological parameter (Fig. 2B).

### 3.3. Differential expression of core targets

**Figure 3.**
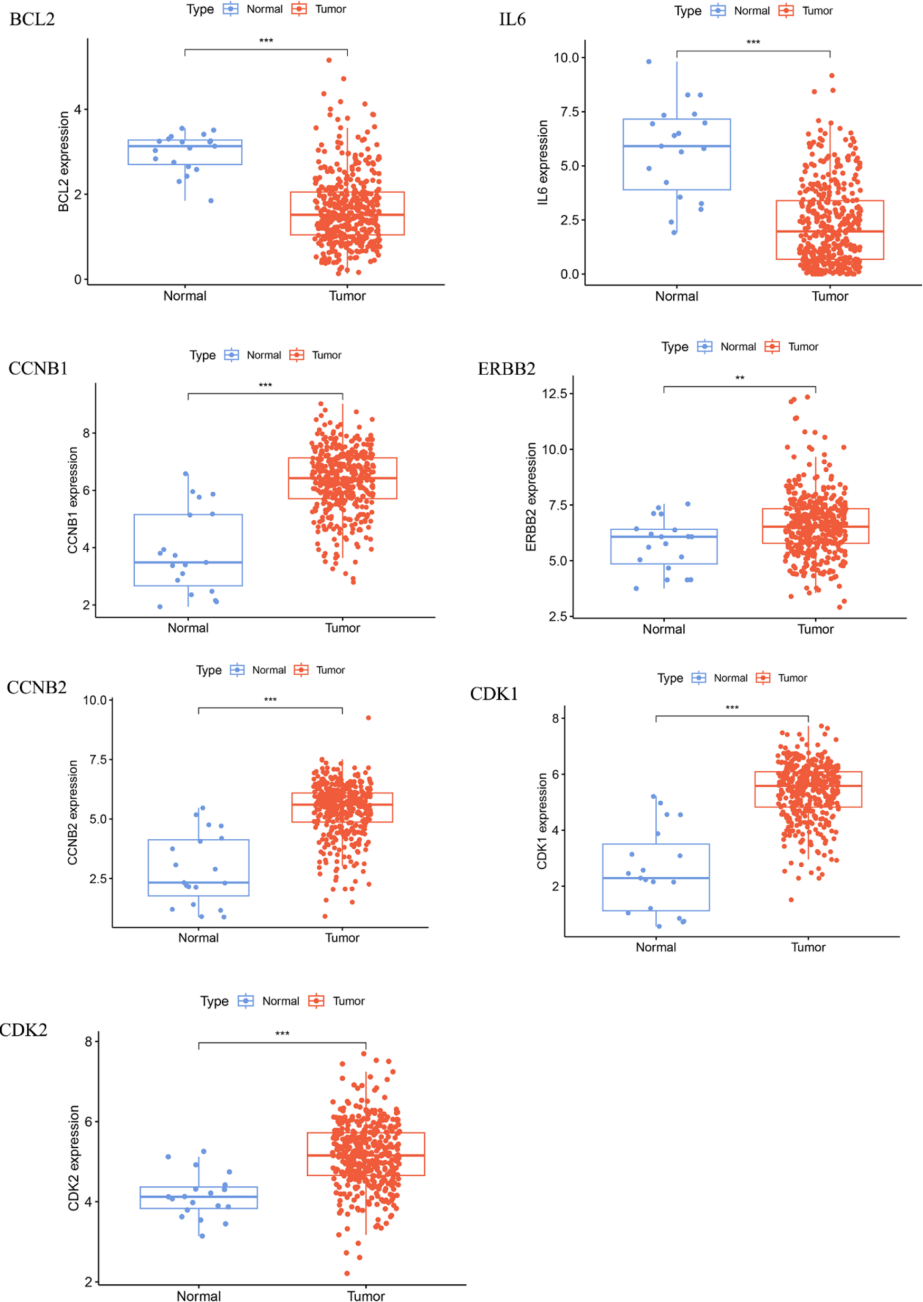
Differential expression of core targets. The above image shows the differences in the expression of core targets between BLCA tissue and normal tissue. Red represents tumor tissue; blue represents normal tissue. * represents a *P*-value < .05, ** represents a *P*-value < .01, *** represents a *P*-value < .001. BLCA = bladder cancer, CCNB1 = cyclin B1, CCNB2 = cyclin B2, CDK1 = cyclin-dependent kinase 1, CDK2 = cyclin-dependent kinase 2, ERBB2 = Erb-B2 receptor tyrosine kinase 2, IL6 = interleukin 6.

The differential expression analysis of the core targets obtained above, performed using R, revealed significant differences in the expression of these targets between tumor and normal tissue samples. Notably, the expression levels of IL6 were significantly downregulated in tumor tissue samples (*P* *<* .05), while BCL2 was significantly downregulated (*P* *<* .01). In contrast, CDK1, CDK2, CCNB1, ERBB2, and CCNB2 were significantly upregulated in tumor tissue samples (*P* *<* .001), all of which demonstrated statistically significant differences. These core targets exhibited consistent patterns of either downregulation or upregulation in tumor tissues, suggesting their potential involvement in the pathogenesis of DEHP-induced BLCA (Fig. 3).

### 3.4. Functional analysis and pathway enrichment analysis of the targets

**Figure 4.**
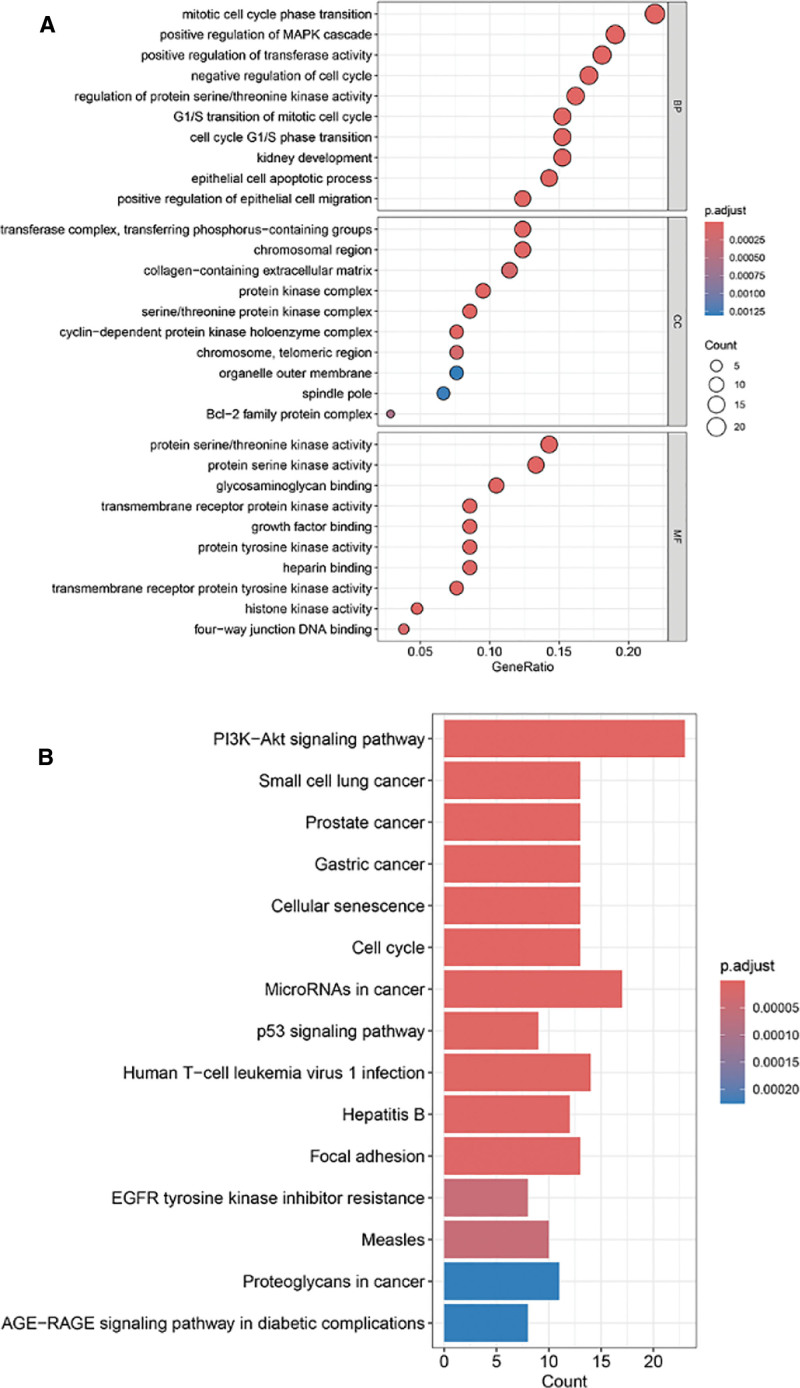
GO and KEGG enrichment analysis of potential targets. (A) The bubble chart shows a GO analysis of 105 potential targets; the size of the circle indicates the number of enriched genes, and the redder and darker the color is, the greater the significance. (B) The histogram shows a KEGG signaling pathways enriched in 105 potential targets, and each bar length represents the number of enriched genes in that pathway. The redder and darker the color, the more significant it is. GO = Gene Ontology, KEGG = Kyoto Encyclopedia of Genes and Genomes.

We conducted GO and KEGG enrichment analyses of 105 potential targets using R. The GO analysis identified 1439 GO terms, including 1339 biological processes, 51 cellular components, and 49 molecular functions. In addition, 61 KEGG signaling pathways were identified. The top 10 biological processes, cellular components, and molecular functions, as well as the top 15 KEGG signaling pathways, were visualized based on the lowest false discovery rate values. Notably, the cellular components primarily showed enrichment in complexes related to cell cycle regulation and signal transduction, such as components of the transferase complex and chromatin regions. The molecular function analysis predominantly highlighted binding activities, including protein serine/threonine kinase activity and transmembrane receptor protein kinase activity, both of which are crucial in the regulation of cell proliferation, differentiation, and signaling pathways. In the biological process analysis, the focus was mainly on cytokinesis, transferase activity, and the MAPK cascade, all of which are essential in regulating cellular responses to external stimuli and promoting cell proliferation (Fig. 4A). In the KEGG signaling pathway enrichment analysis, significant enrichment was observed in cancer-related pathways, including the PI3K-Akt signaling pathway, p53 signaling pathway, and cancer-associated microRNAs, which are well-established in their roles in cell proliferation, differentiation, migration, and invasion (Fig. 4B).

### 3.5. Pathway enrichment analysis of core targets

**Figure 5.**
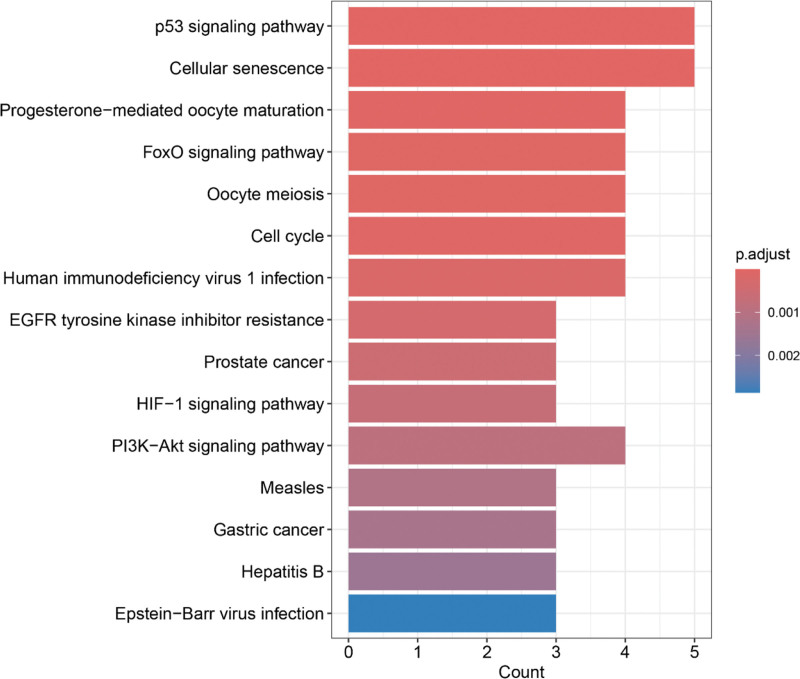
KEGG enrichment analysis of core targets. The histogram shows a KEGG signaling pathways enriched at the 7 core targets. KEGG = Kyoto Encyclopedia of Genes and Genomes.

We conducted KEGG enrichment analyses using the R programming language for the 7 core targets, which resulted in the identification of 27 enriched pathways. The top 15 KEGG signaling pathways were visualized according to the lowest false discovery rate values. Notably, these core targets were significantly enriched in cancer-related pathways, such as the PI3K-Akt signaling pathway and the p53 signaling pathway, further supporting the consistency of our findings with the type of BLCA induced by DEHP (Fig. 5).

### 3.6. Molecular docking of DEHP and core target proteins

**Figure 6.**
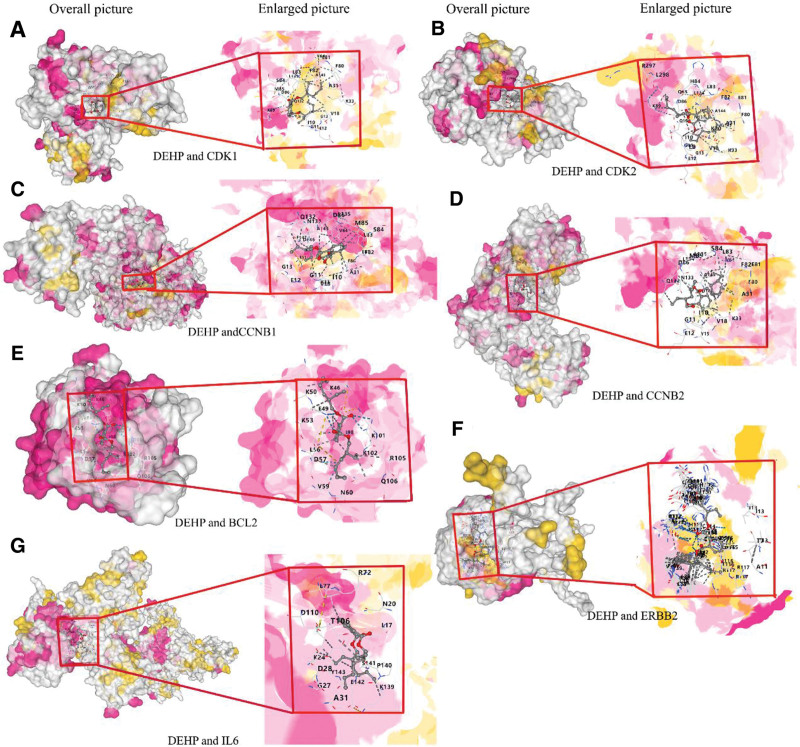
DEHP-induced molecular docking node results for each core target of BLCA. (A) DEHP and CDK1, (B) DEHP and CDK2, (C) DEHP and CCNB1, (D) DEHP and CCNB2, (E) DEHP and BCL2, (F) DEHP and ERBB2, (G) DEHP and IL6. BCL2 = B-cell lymphoma 2, BLCA = bladder cancer, CCNB1 = cyclin B1, CCNB2 = cyclin B2, CDK1 = cyclin-dependent kinase 1, CDK2 = cyclin-dependent kinase 2, DEHP = di(2-ethylhexyl) phthalate, ERBB2 = Erb-B2 receptor tyrosine kinase 2, IL6 = interleukin 6.

Molecular docking results revealed that DEHP tightly binds to CDK1, IL6, CDK2, CCNB1, ERBB2, CCNB2, and BCL2, with binding energies <5 kcal/mol (−6.9, −5.5, −7.6, −7.9, −22.3, −8.1, and −5.6 kcal/mol, respectively). This suggests that the binding stability between DEHP and each target is high. DEHP interacts with these targets through hydrogen bonds, ionic bonds, and other interactions, demonstrating strong binding forces (Fig. 6). These findings indicate that the tight binding of DEHP to these coreceptors under long-term exposure conditions may play a crucial role in the molecular mechanism of BLCA.

## 4. Discussion

In this study, we systematically identified 105 potential targets associated with DEHP-induced BLCA through the ChEMBL, STITCH, SwissTargetPrediction, GeneCards, OMIM, and TTD databases. Based on the STRING and Cytoscape platforms, we constructed a PPI network and identified 7 core targets: CDK1, IL6, CDK2, CCNB1, ERBB2, CCNB2, and BCL2.

Cyclins and cyclin-dependent kinases are key regulators of the cell cycle, and any abnormalities in either can lead to cell cycle dysregulation. Members of the CDK family must be activated by the expression of cyclins to regulate the eukaryotic cell cycle. CCNB2 and CCNB1 are B-type cyclins, while CDK1 and CDK2 are members of the cyclin-dependent kinase family. It has been reported that the downregulation of CCNB2 expression in BLCA inhibits cancer cell invasion and metastasis.^[30]^ CCNB1 is a major regulator of the G2/M transition, with its expression peaking during mitosis.^[31]^ CCNB1 levels accumulate significantly during the G2/M cell cycle transition and interact with CDK1 to form a complex, promoting the G2/M transition in the cell cycle.^[32]^ Previous research indicates that inactivation of p53 results in CCNB1 overexpression, contributing to tumor progression.^[33]^ Studies have reported that in BLCA, PBRM1 inhibits CCNB1 expression, blocking the G2/M transition and exerting a tumor-suppressive effect.^[34]^

Both CDK1 and CDK2 are highly expressed in various malignancies.^[35]^ Consistent with the findings of this study, the phosphorylation of CDK1 at the Thr177 site plays a crucial role in BLCA pathogenesis, where CDK1 phosphorylates Tfcp2 11 at the same site, influencing cell cycle progression, multicellularity, and embryonic stem cell differentiation. The activation of the CDK1-TFCP2L1 pathway has been demonstrated to stimulate cancer cell proliferation, self-renewal, and invasion.^[36]^ Moreover, the interaction of CDK1 with Cyclin B1 has been reported to facilitate the G2/M phase transition, thereby promoting BLCA cell proliferation.^[37]^ Studies have indicated that CDK2 enhances cell growth by modulating the cell cycle. For example, MTHFD2 has been shown to activate CDK2 to promote BLCA cell proliferation.^[38]^

IL-6, a member of the IL-6 family, plays a role in maintaining immune homeostasis, hematopoiesis, inflammation, development, and metabolism. Consequently, abnormalities in IL-6 activity are often associated with chronic diseases and cancer development.^[39]^ For example, IL-6 secretion promotes the proliferation and stemness of UBC cells.^[40]^ Moreover, IL6 has been identified as a predictor of lymph node metastasis in BLCA patients and is markedly elevated in the serum of affected patients.^[41]^

ERBB2, also known as HER2, is a receptor tyrosine kinase that belongs to the EGFR/ERBB/HER kinase family. It is mutated or amplified in various types of cancer. ERBB2 plays a crucial role in cell transformation and tumourigenesis mediated by its mutations.^[42]^ ERBB2 activation and mutation have been implicated in muscle-invasive BLCA.^[43]^ Studies have demonstrated that ERBB2 is highly expressed in BLCA cells and is associated with distant tumor metastasis through CRK mechanisms.^[44]^ Patients with HER2 overexpression are at the highest risk for disease recurrence and progression after exposure to Bacillus Calmette–Guérin treatment.^[45]^

BCL2 is an antiapoptotic cancer protein that inhibits cell death by preventing the release of cytochrome c from the mitochondria and activation of caspases.^[46]^ Downregulation of BCL-2 induces Caspase-3-mediated apoptosis and inhibits the classic PI3K/Akt/mTOR signaling pathway, thereby suppressing the proliferation and migration of BLCA cells.^[47]^ It has been reported that BCL2 is highly methylated in the cancerous tissues and urine of BLCA patients, and the methylation level correlates with tumor grade and stage.^[48]^

In conclusion, our findings suggest that DEHP exposure may significantly influence the incidence of BLCA through its interactions with these identified targets, underscoring the need for further investigation into the molecular mechanisms underlying DEHP-induced carcinogenesis in bladder tissues.

To gain deeper insights into the biological mechanisms of DEHP exposure, this study performed GO and KEGG pathway enrichment analyses on 105 potential target genes, revealing biological pathways potentially affected by DEHP exposure. GO analysis indicated that the target genes are involved in cellular processes such as cell cycle regulation and signal transduction. KEGG pathway enrichment analysis showed that DEHP exposure may influence BLCA progression through various toxic pathways, including the PI3K-Akt and P53 signaling pathways. These pathways are involved in processes such as inflammation, apoptosis, tumor microenvironment, angiogenesis, metabolic alterations, and immune regulation, all of which could contribute to the initiation and progression of BLCA.

The PI3K/Akt pathway is critically involved in cell survival and proliferation.^[49]^ In this pathway, Akt activation drives glycolysis and lactate production, supporting tumor survival under hypoxic conditions.^[50]^ The activation of the PI3K/Akt pathway is crucial for the onset and progression of BLCA,^[51]^ likely by inhibiting apoptosis and promoting cell survival and proliferation, leading to BLCA development.

Under physiological conditions, various stress signals activate the p53 signaling pathway, leading cells to initiate multiple transcriptional programmes, including cell cycle arrest, DNA repair, senescence, and apoptosis, thereby effectively inhibiting tumor growth.^[52]^ Lim et al^[53]^ discussed how TP53 inactivation initiates tumorigenesis, contributes to treatment resistance, promotes migration, and facilitates metastasis. Moreover, mutated p53 proteins may disrupt autophagic processes, exacerbate antiapoptotic effects, and promote the proliferation of cancer cells.^[54]^ Zhang and Zhang reported the detection of p53 gene mutations in urine samples from bladder cancer patients, emphasizing the potential of p53 mutations as biomarkers for diagnosis and prognosis.^[55]^

Molecular docking results revealed that 7 core target proteins stably bind to the active pocket of DEHP, with binding energies below 5 kcal/mol, confirming their significant roles in DEHP-induced BLCA.

Although network toxicology-based approaches have yielded valuable insights into the molecular mechanisms of DEHP-induced BLCA, this study is subject to several limitations. Our investigation was primarily reliant on computational modeling, which, while beneficial for generating important insights, does not fully capture the complexities of long-term and low-dose exposure scenarios that humans may encounter with microplastics. Typically, animal studies utilize high-dose, short-term exposure protocols, which may not adequately simulate chronic, low-level exposures from consumer products and environmental contamination. By applying network toxicology to the study of microplastics, researchers can rapidly identify key molecular targets, thereby assisting in the formulation of more effective regulatory standards and preventive measures to mitigate the health risks associated with environmental contaminants such as DEHP.

Furthermore, cyber toxicology offers a rapid and comprehensive approach for evaluating the toxicity of emerging chemicals by incorporating bioinformatics, multi-omics, and big data analytics. Using molecular docking techniques has improved the predictive accuracy of toxicological evaluations, offering an effective way to clarify intricate molecular interactions. Although this study presents promising results, additional research is needed to confirm the key molecular targets and pathways identified through extensive epidemiological studies and animal models that accurately mimic chronic, low-dose DEHP exposure. These investigations provide a robust theoretical foundation for the development of preventive and therapeutic strategies to address DEHP-induced health risks associated with BLCA.

## 5. Conclusion

In conclusion, this study employed network toxicology and molecular docking techniques to explore the potential molecular mechanisms of DEHP exposure in BLCA. By integrating data from multiple databases and constructing a PPI network, we identified 7 key core targets in BLCA: CDK1, IL6, CDK2, CCNB1, ERBB2, CCNB2, and BCL2. Enrichment analysis revealed that the target genes were significantly associated with the PI3K-Akt and p53 signaling pathways, which are involved in cell proliferation, differentiation, and apoptosis. Furthermore, the integration of TCGA data with other databases enhanced the precision of the analysis. This study uncovers the potential molecular mechanisms by which DEHP exposure induces BLCA through multi-omics pathways. It also provides a standardized framework for using network toxicology to study the effects of environmental pollutants.

## Acknowledgments

The authors would like to express sincere gratitude to the Natural Science Foundation of Hainan Province for their support (Grant No. 822RC842).

## Author contributions

**Conceptualization:** Chuanwei Sun, Qianghua Hu.

**Data curation:** Baofeng Wang, Qingmai Huang.

**Funding acquisition:** Cianping Che.

**Methodology:** Manfei Jiang.

**Supervision:** Xianping Che.

**Writing – original draft:** Manfei Jiang.

**Writing – review & editing:** Chuanwei Sun.
